# Lack of satellite DNA species-specific homogenization and relationship to chromosomal rearrangements in monitor lizards (Varanidae, Squamata)

**DOI:** 10.1186/s12862-017-1044-6

**Published:** 2017-08-16

**Authors:** Ornjira Prakhongcheep, Watcharaporn Thapana, Aorarat Suntronpong, Worapong Singchat, Khampee Pattanatanang, Rattanin Phatcharakullawarawat, Narongrit Muangmai, Surin Peyachoknagul, Kazumi Matsubara, Tariq Ezaz, Kornsorn Srikulnath

**Affiliations:** 1 Laboratory of Animal Cytogenetics and Comparative Genomics (ACCG), Department of Genetics, Faculty of Science, Kasetsart University, 50 Ngamwongwan, Chatuchak, Bangkok, 10900 Thailand; 2 Animal Breeding and Genetics Consortium - Kasetsart University (ABG - KU), 50 Ngamwongwan, Chatuchak, Bangkok, 10900 Thailand; 3 Center for Advanced Studies in Tropical Natural Resources, National Research University-Kasetsart University (CASTNAR, NRU-KU), Kasetsart University, Bangkok, 10900 Thailand; 4 Department of Parasitology, Faculty of Veterinary Medicine, Kasetsart University, 50 Ngamwongwan, Chatuchak, Bangkok, 10900 Thailand; 5 Real Zoo, The Sky Shopping Center, Ayutthaya, 13210 Thailand; 6 Mildpets Animal Hospital, 169/10-11 Keharomkloa 31 Road, Klongsongtonnun, Ladkrabang, Bangkok, 10520 Thailand; 7 Department of Fishery Biology, Faculty of Fisheries, Kasetsart University, 50 Ngamwongwan, Chatuchak, Bangkok, 10900 Thailand; 8 Department of Biology, Faculty of Science, Naresuan University, Phitsanulok, 65000 Thailand; 9 Wildlife Genetics Laboratory, Institute for Applied Ecology, University of Canberra, Canberra, ACT 2600 Australia

**Keywords:** Nucleotide sequence conservation, Repeated sequence, Lizard, Homogenization, Macrochromosome

## Abstract

**Background:**

Satellite DNAs (stDNAs) are highly repeated sequences that constitute large portions of any genome. The evolutionary dynamics of stDNA (e.g. copy number, nucleotide sequence, location) can, therefore, provide an insight into genome organization and evolution. We investigated the evolutionary origin of VSAREP stDNA in 17 monitor lizards (seven Asian, five Australian, and five African) at molecular and cytogenetic level.

**Results:**

Results revealed that VSAREP is conserved in the genome of Asian and Australian varanids, but not in African varanids, suggesting that these sequences are either differentiated or lost in the African varanids. Phylogenetic and arrangement network analyses revealed the existence of at least four VSAREP subfamilies. The similarity of each sequence unit within the same VSAREP subfamily from different species was higher than those of other VSAREP subfamilies belonging to the same species. Additionally, all VSAREP subfamilies isolated from the three Australian species (*Varanus rosenbergi*, *V. gouldii*, and *V. acanthurus*) were co-localized near the centromeric or pericentromeric regions of the macrochromosomes, except for chromosomes 3 and 4 in each Australian varanid. However, their chromosomal arrangements were different among species.

**Conclusions:**

The VSAREP stDNA family lack homogenized species-specific nucleotide positions in varanid lineage. Most VSAREP sequences were shared among varanids within the four VSAREP subfamilies. This suggests that nucleotide substitutions in each varanid species accumulated more slowly than homogenization rates in each VSAREP subfamily, resulting in non-species-specific evolution of stDNA profiles. Moreover, changes in location of VSAREP stDNA in each Australian varanid suggests a correlation with chromosomal rearrangements, leading to karyotypic differences among these species.

**Electronic supplementary material:**

The online version of this article (doi:10.1186/s12862-017-1044-6) contains supplementary material, which is available to authorized users.

## Background

Whole genome sequencing technology is applied to both coding and non-coding sequences in vertebrates, though the assembly process is still complicated for repeated non-coding sequences, even in the centromeric region [1–3], with a possible knowledge gap in elucidating their function and evolution [4, 5]. Repeated sequences are commonly characterized into two main classes: the site-specific type (such as satellite DNA, microsatellite repeats, ribosomal RNA genes, and telomeric sequences), and the interspersed type (transposable elements). A large fraction of site-specific repetitive sequences is composed of tandem repeated sequences known as satellite DNA (stDNA), mostly located at the heterochromatic regions of chromosomes as centromeres and telomeres [6–8]. The stDNAs are considered to be involved in the organization of chromosomes during mitosis or meiosis; they are also genomic elements which differentiate rapidly within the genome [9, 10]. Multiple stDNA families of independent origin coexist in the genome of a species, and they commonly differ in nucleotide sequences and copy number [9, 11–15]. Within a species, monomers of a stDNA family may exhibit higher sequence similarity than the same stDNA family of related species [9, 16, 17]. This indicates that mutations in stDNA monomers are homogenized and concomitantly fixed in a group of reproductively linked species [16, 18–21]. This phenomenon varies among stDNA families based on mutation rate, chromosome morphology and distribution, population size and genetic drift, divergence time, and reproductive mode [22–27]. However, the process of stDNA differentiation occurred rapidly among species, leading to the expansion of new mutations horizontally throughout the genome [10]. Therefore, stDNA sequences can also be used as phylogenetically informative markers shared among diverse lineages [10, 14, 28, 29]. Simultaneously, stDNAs are thought to play an important role in chromosome evolution, in which they appear to act as a substrate for homologous or non-homologous recombination resulting in chromosomal rearrangements [2, 30, 31].

Monitor lizards or varanids comprise a single extant genus, *Varanus*, within the family Varanidae. Currently, 79 extant species are described and they are distributed in Afro-Arabia, Western to Southeast Asia, the Indonesian Archipelago, Papua New Guinea, and Australia [32]. The diploid chromosome number of most varanids is 40, comprising 16 macro- and 24 microchromosomes. The karyotypic differentiation in several varanids is based on changes involving macrochromosome morphology [7, 33–38]. Importantly, the karyotype of at least five varanids (*Varanus salvator macromaculatus*, *V. acanthurus*, *V. gouldii*, *V. rosenbergi*, and *V. komodoensis*) comprises large C-positive heterochromatin blocks that are considered to contain many repeated sequences at the centromeric or pericentromeric regions of both macro- and microchromosomes, and the distal region of chromosome 1q [7, 34, 36, 38]. The characterization of repeated sequences is thus necessary for a better understanding of genome organization and chromosome evolution in the varanid lineage. The centromeric VSAREP stDNA family was isolated from an Asian varanid (*V. salvator macromaculatus*). This was not found in other squamate reptiles, including the African varanid (*V. exanthematicus*) [7]. By contrast, stDNA families isolated from lacertid lizards and snakes are widely conserved at family level [8, 39–44]. There may be a broad taxonomic distribution of VSAREP in varanid lineages, and analyses of such sequences in additional varanids are required to provide more conclusive evidence of their evolutionary origin, diversification, and relation to chromosomal changes. This study investigated the presence of VSAREP in 17 varanids (seven Asian, five Australian, and five African) using dot-blot hybridization. Various DNA fragments of VSAREP were cloned from Asian and Australian varanids to determine their nucleotide sequences and substitution rates. Chromosomal distribution of VSAREP stDNA was examined in three Australian varanids (*V. rosenbergi*, *V. gouldii*, and *V. acanthurus*). The evolutionary dynamics of repeated sequence families are also discussed.

## Methods

### Animals and DNA extraction

Seventeen varanids (both species and subspecies) were examined, and detailed information including abbreviation, biogeography, sex, and location regarding these individuals is presented in Table 1. Blood was used as source of DNA and was collected from the ventral caudal vein using a 25-gauge needle attached to a 1 ml disposable syringe containing 10 mM ethylenediaminetetraacetic acid (EDTA). Whole genomic DNA was extracted following the standard salting-out protocol as described previously [45]. DNA quality and quantity were determined using 1% agarose gel electrophoresis and spectrophotometric analysis. Animal care and all experimental procedures were approved by the Animal Experiment Committee, Kasetsart University, Thailand (approval no. ACKU59-SCI-006) and the University of Canberra, Australia (permit no. CEAE 11/07), and conducted according to the Regulations on Animal Experiments at both Universities.

**Table 1:** Summary of repeat features and nucleotide diversity (π values) for each species used in this study

Species	Abbreviation	Biogeography	Sex	Locations	n	Repeat length (bp)	%GC	Nucleotide diversity (π)	Accession number
*Varanus salvator macromaculatus*	VSA(M)	Asia	Male	Nakhon Ratchasima Zoo (Thailand)	10	185–190	58.90 and 60.50	0.10379 ± 0.00788	LC190690–LC190699
*Varanus salvator sulfur*	VSA(S)	Asia	Unknow	Real Zoo (Thailand)	14	188–192	58.80	0.11218 ± 0.00699	LC190700–LC190713
*Varanus salvator ziegleri*	VSA(Z)	Asia	Male	Real Zoo (Thailand)	6	189–206	58.40	0.10303 ± 0.01617	LC190714–LC190719
*Varanus bengalensis*	VBE	Asia	Unknow	Nakhon Ratchasima Zoo (Thailand)	16	176–191	55.20	0.16355 ± 0.01432	LC190514–LC190529
*Varanus nebulosus*	VNE	Asia	Unknow	Nakhon Ratchasima Zoo (Thailand)	9	187–196	59.80	0.12168 ± 0.01102	LC190604–LC190612
*Varanus rudicollis*	VRU	Asia	Unknow	Nakhon Ratchasima Zoo (Thailand)	21	185–191	61.90	0.11866 ± 0.00883	LC190669–LC190689
*Varanus dumerilii*	VDU	Asia	Male	Real Zoo (Thailand)	20	183–188	58.90	0.21252 ± 0.01785	LC190530–LC190549
*Varanus salvadorii*	VSALV	Australia	Unknow	Real Zoo (Thailand)	2	191	54.70	0.26455 ± 0.13228	LC190720–LC190721
*Varanus komodoensis*	VKO	Australia	Unknow	Real Zoo (Thailand)	21	188–191	57.60	0.15862 ± 0.00915	LC190583–LC190603
*Varanus rosenbergi*	VRO	Australia	Male and Female	NSW, Australia, (private breeders)	56	189–192	59.80 and 59.20^a^	0.14456 ± 0.00503	LC190613–LC190668
*Varanus gouldii*	VGO	Australia	Male and Female	NSW, Australia, (private breeders)	33	183–192	60.20	0.09411 ± 0.00590	LC190550–LC190582
*Varanus acanthurus*	VAC	Australia	Male and Female	NT, Australia, (private breeders)	13	189–194	61.00 and 58.20^a^	0.15780 ± 0.02191	LC185194–LC185206
*Varanus exanthematicus*	VEX	Africa	Male	Real Zoo (Thailand)	-	-	-	-	-
*Varanus niloticus*	VNI	Africa	Female	Real Zoo (Thailand)	-	-	-	-	-
*Varanus jobiensis*	VJO	Africa	Female	Real Zoo (Thailand)	-	-	-	-	-
*Varanus obor*	VOB	Africa	Unknow	Real Zoo (Thailand)	-	-	-	-	-
*Varanus griseus*	VGR	Africa	Unknow	Real Zoo (Thailand)	-	-	-	-	-

Number of monomeric repeats sequenced (n), nucleotide composition of repeats (GC), length of repeats, and nucleotide diversity (π) ± SD of each varanid

^a^Nucleotide composition of repeats were derived from different repeated subfamilies

### Dot-blot hybridization

Dot-blot hybridization was performed to examine the conservation of VSAREP repeated sequences among the 16 different varanids, except for *V. rosenbergi* (VRO) due to insufficient amount of DNA. To prepare the dot-blots, 200 ng of genomic DNA was denatured with 0.4 N NaOH for 10 min and then transferred onto nylon membrane. DNA fragments of repeated sequences (VSAREP1 or VSAREP2) derived from pFOSVSA1 and pFOSVSA2 clones in the previous study [7] were labeled with DIG-11-dUTP using PCR DIG Labeling Mix (Roche Diagnostics, Indianapolis, IN, USA) and universal M13 primers (M13F-pUC (−40): 5′-GTTTTCCCAGTCACGAC-3′ and M13R (−20): 5′-GCGGATAACAATTTCACACAGG-3′) according to the manufacturer’s instructions and hybridized to the membranes at 45 °C overnight in DIG Easy Hyb solution (Roche Diagnostics). After hybridization, the membranes were washed at 45 °C in 0.1% sodium dodecyl sulfate (SDS)/2× saline-sodium citrate (SSC), 0.1% SDS/1× SSC, 0.1% SDS/0.5× SSC, and 0.1% SDS/0.1× SSC for 15 min each. Chemiluminescent signals were detected using anti-digoxigenin-AP Fab fragments and CDP-Star (Roche Diagnostics) and exposed to KODAK T-MAT G/RA dental film (Carestream Health, Rochester, NY, USA).

### Molecular cloning and sequence analysis

DNA fragments of VSAREP stDNA sequences were amplified using target-specific primers VSA1-F: 5′-CGGCACCCTTCCAGACTC-3′ and VSA1-R: 5′- GCCAGAAAAGTCTGTCCAAAATGC-3′, which were designed based on VSAREP sequences (accession numbers: AB773867 and AB773868) [7]. PCR amplification was performed using 15 μl of 1× ThermoPol buffer containing 1.5 mM MgCl_2_, 0.2 mM dNTPs, 5.0 μM of primers, 0.5 U of *Taq* polymerase (Vivantis Technologies Sdn Bhd, Selangor Darul Ehsan, Malaysia), and 25 ng of genomic DNA. PCR conditions were as follows: an initial denaturation at 94 °C for 3 min, followed by 35 cycles of 94 °C for 30 s, 52 °C for 40 s, and 72 °C for 1 min 30 s, and a final extension at 72 °C for 10 min. PCR products were visualized by electrophoresis on 1% agarose gel. PCR product sizes between 190 and 760 bp were molecularly cloned using the pTG19-T cloning vector (Vivantis Technologies Sdn Bhd), and the nucleotide sequences of the DNA fragments were determined using the DNA sequencing services of First BASE Laboratories Sdn Bhd (Seri Kembangan, Selangor, Malaysia). Individual monomers were then identified within multimers. Nucleotide sequences of at least two DNA clones in each varanid were searched for homologies using the BLASTn program (http://blast.ncbi.nlm.nih.gov/Blast.cgi). Additionally, the nucleotide sequence was searched for regions which formed characteristic secondary structures using RNAfold web server (http://rna.tbi.univie.ac.at/cgi-bin/RNAWebSuite/RNAfold.cgi) [46]. Multiple sequence alignment was performed with multiple sequence comparison by log-expectation (MUSCLE) (http://www.ebi.ac.uk/Tools/msa/muscle/) [47], using default parameters. After visual inspection of alignments, sequences were identified into a repeated unit and then deposited in the DNA Data Bank of Japan (DDBJ; http://www.ddbj.nig.ac.jp/index-e.html) (Table 1). Intraspecific nucleotide diversity (π value) and stDNA subfamily diversity were estimated using DnaSP v. 5 [48]. Numbers of insertions and deletions (indels) were manually calculated for each repeated unit of all species. A consensus sequence based on the total alignment of units in each stDNA subfamily of species was constructed using BioEdit sequence alignment editor version 7.2.5 [49] by choosing the most frequent nucleotide at each position. The level of sequence divergence between the species or between stDNA subfamily was estimated using uncorrected pairwise distances (*p*-distances) as implemented in MEGA6 [50]. Phylogenetic analysis was then performed, using Bayesian inference (BI) with MrBayes v3.0b4 [51]. The Markov chain Monte Carlo process was used to run four chains simultaneously for one million generations, sampling every 100 generations. Log likelihood and parameter values were assessed with Tracers ver. 1.5 [52]. A burn-in of 25% of saved trees was removed, and the remaining trees were used to generate a majority-rule consensus tree with average branch lengths. The Bayesian posterior probability in the sampled tree population was obtained in percentage terms. A phylogenetic network of the consensus sequences was constructed using statistical parsimony generated in PopART v1.7. AMOVA [53] was used to detect genetic differentiation among stDNA sequences by determining molecular variance and calculating F-statistics using ARLEQUIN 2.000 with 1000 permutations [54]. This was performed at two hierarchical levels to test how stDNA sequence variability was distributed both within and among the varanids analyzed (species and subspecies level) and within and among stDNA subfamilies detected.

### Fluorescence in situ hybridization (FISH) mapping

The chromosomal location of two VSAREP stDNA sequences (VSAREP1 and VSAREP2) was determined in three Australian varanids using two color FISH, as described previously [55]. Chromosomes of these species were prepared in previous studies [36, 37]. Two 40-kb genomic DNA fragments of VSAREP1 and VSAREP2 containing all repeated units in each fragment were derived using pFOSVSA1 and pFOSVSA2 clones from *V. salvator macromaculatus* in the previous study [7]. Approximately, 250 ng of 2 repeated DNA fragments were labeled separately by nick translation incorporating SpectrumGreen-dUTP (Abbott, North Chicago, Illinois, USA) or SpectrumOrange-dUTP (Abbott). Each labeled probe was precipitated with 20 μg glycogen as carrier and dissolved in 15 μl hybridization buffer. Then, 12.5 μl of the hybridization mixture was placed on a chromosome slide and sealed with a coverslip and rubber cement. Probe DNA and chromosome DNA were denatured simultaneously by heating the slide on a heat plate at 68.5 °C for 5 min. The slides were hybridized overnight in a humidified chamber at 37 °C. They were then washed once following the series: 0.4× SSC, 0.3% IGEPAL (Sigma-Aldrich) at 55 °C for 2 min followed by 2× SSC, 0.1% IGEPAL at room temperature for 1 min. The slides were dehydrated through an ethanol series, air-dried and then counterstained using 20 mg/ml DAPI (4′,6-diamidino-2-phenylindole), 2× SSC and mounted with antifade medium Vectashield (Vector Laboratories, Burlingame, California, USA).

The chromosomal locations of VSAREP isolated from genomic DNA of each Australian varanid were determined using FISH or two color FISH with randomly selected VSAREP clones from each stDNA subfamily in which nucleotide sequences were determined (Table 1) as described previously [56, 57]. Approximately 250 ng of stDNA fragments were labeled separately, incorporating biotin-16-dUTP (Roche Diagnostics) or digoxigenin-11-dUTP (Roche Diagnostics) by nick translation according to the manufacturer’s protocol (Additional file 1: Table S1). After hybridization, probes were detected by incubating the chromosome slides with anti-digoxigenin-rhodamine Fab fragments (Roche Diagnostics) and avidin labeled with fluorescein isothiocyanate (avidin-FITC; Invitrogen, CA, USA), respectively. Slides were counter-stained with 1 μg/ml DAPI. Fluorescence hybridization signals were captured using a cooled CCD camera mounted on a ZEISS Axioplan 2 microscope and analyzed using MetaSystems ISIS v.5.2.8 software (MetaSystems, Alltlussheim, Germany).

## Results

### Dot-blot analysis

**Fig. 1:**
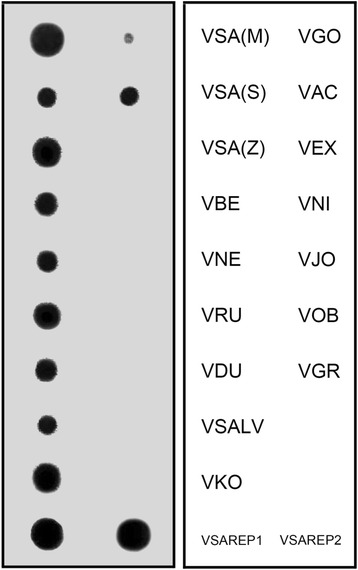
Dot-blot hybridization probed with VSAREP1. Genomic DNAs of 16 varanids were used: *Varanus salvator macromaculatus* (VSA(M)), *V. salvator sulfur* (VSA(S)), *V. salvator ziegleri* (VSA(Z)), *V. bengalensis* (VBE), *V. nebulosus* (VNE), *V. rudicollis* (VRU), *V. dumerilii* (VDU), *V. salvadorii* (VSALV), *V. komodoensis* (VKO), *V. gouldii* (VGO), *V. acanthurus* (VAC), *V. exanthematicus* (VEX), *V. niloticus* (VNI), *V. jobiensis* (VJO), *V. obor* (VOB), and *V. griseus* (VGR). Clones VSAREP1 and VSAREP2 were used as control. Intense hybridization signals were observed for all Asian and Australian varanids; however, no signal was detected in the five African varanids

Conservation of VSAREP1 was examined by dot-blot hybridization of 16 varanids, except for *V. rosenbergi*, using their genomic DNA. Intense hybridization signals were observed for all Asian and Australian varanids; however, no signal was detected in the five African varanids (Fig. 1). Similar results were found for the hybridization of VSAREP2 (data not shown).

### Isolation and characterization of VSAREP stDNA family

Specific VSAREP primers were used to amplify VSAREP sequences in 16 varanids, except for *V. salvator macromaculatus*. After gel electrophoresis, PCR products showed a ladder-like pattern of DNA bands typical of stDNAs in all Asian and Australian varanids, but not in African varanids (data not shown). This pattern was based on the repetition of the 185–190 bp monomer unit. In addition to the five sequences of each VSAREP1 and VSAREP2 isolated from *V. salvator macromaculatus* in our previous study [7], a total of 211 new sequences of monomer units were obtained with length ranging from 176 to 206 bp. Several indels from 1 to 14 bp were detected. All VSAREP sequences were GC-rich (average GC content of 57.27%) and characterized by possessing a secondary structure (Additional file 2: Figure S1). The conserved sequence motifs of VSAREP stDNA family as “TGACCCGCGGGTCAGC” and “TTTTBGGCATTTTG” were found in all sequence units (Additional file 3: Figure S2). BLASTn search of all VSAREP sequence units showed similarity ranging from 54.50% (*V. dumerilii*) to 97.60% (*V. salvator ziegleri*) with VSAREP1 and VSAREP2. No significant similarity was found with other sequences deposited in databases.

**Fig. 2:**
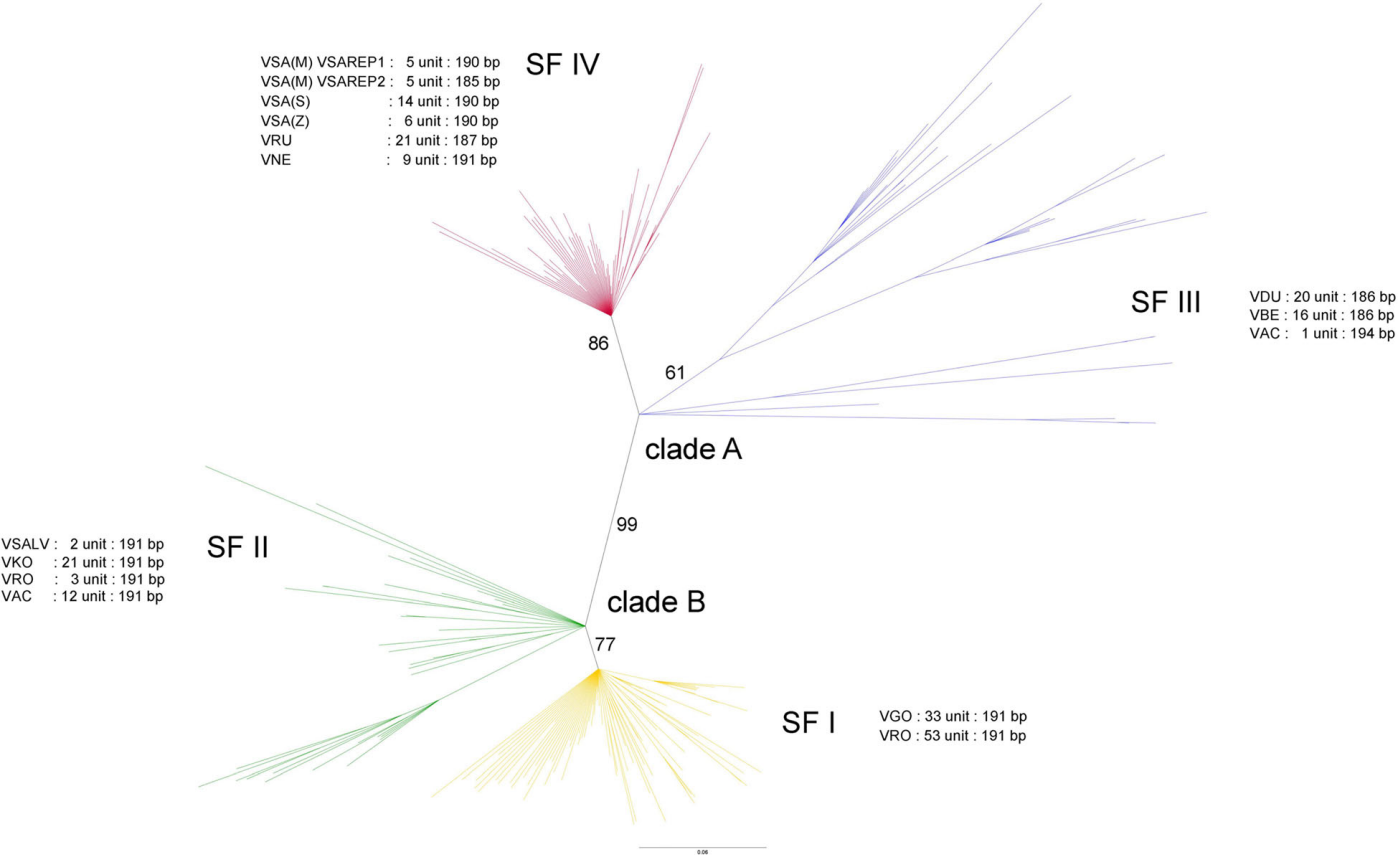
Phylogenetic relationships of VSAREP satellite DNA sequences among 12 varanids inferred using Bayesian inference analysis. Support values at each node are Bayesian posterior probability. A colored line indicates different subfamilies (VSAREP subfamily I (SFI), SFII, SFIII, and SFIV). VSAREP stDNA sequences of the 12 varanids were: *Varanus salvator macromaculatus* (VSA(M)), *V. salvator sulfur* (VSA(S)), *V. salvator ziegleri* (VSA(Z)), *V. bengalensis* (VBE), *V. nebulosus* (VNE), *V. rudicollis* (VRU), *V. dumerilii* (VDU), *V. salvadorii* (VSALV), *V. komodoensis* (VKO), *V. gouldii* (VGO), *V. acanthurus* (VAC), and *V. rosenbergi* (VRO). All repeated units were grouped together with two major different clades (**a** and **b**). Clade **a** contained SFIII and SFIV, and clade **b** contained SFI and SFII

A Bayesian unrooted phylogenetic tree was constructed to infer the evolutionary relationship between the VSAREP sequences from all varanids and identify putative VSAREP subfamilies. Most monomers were clustered as non-species-specific, but all repeated units were grouped together with two major clades (A and B) of sequences under Asian and Australian varanids. Clade A contained 96 Asian varanid clones with only one clone from *V. acanthurus*, and the other sequences (clade B) consisted of 124 Australian varanid clones (Fig. 2). Clade B contained two VSAREP subfamilies (SFI and SFII), repeated clones from *V. gouldii* and *V. rosenbergi* were found in SFI (38.91% of all clones), while repeat clones from *V. rosenbergi*, *V. acanthurus*, *V. komodoensis*, and *V. salvadorii* were grouped with SFII (17.20%). Clade A contained SFIII (16.74%) and included *V. dumerilii*, *V. bengalensis*, and one clone from *V. acanthurus*, while all repeated clones in SFIV (27.15%) were grouped with Asian varanids (*V. nebulosus*, *V. rudicollis*, *V. salvator sulfur*, *V. salvator macromaculatus*, and *V. salvator ziegleri*) (Additional file 4: Table S2).

### Sequence variability of VSAREP stDNA family within and between species

The average intraspecific sequence divergence (π value) was 14.63% (9.41% ± 0.00590 in *V. gouldii* to 26.46% ± 0.13228 in *V. salvadorii*) (Table 1), whereas the average interspecific sequence divergence (*p*-distance) was 9.35% (0.00% between *V. salvator sulfur* and *V. nebulosus* to 15.60% between *V. gouldii* and *V. bengalensis*) (Additional file 5: Table S3). AMOVA analysis of the VSAREP sequences showed 46.56% intra-species variation (*P* < 0.001) (14.72 of variance components) and 53.44% inter-species variation (*P* < 0.001) (16.89 of variance components).

### Sequence variability of VSAREP stDNA family within and between VSAREP subfamilies

The average π value of each VSAREP subfamily was 13.00% ± 0.0040 for SFI, 20.44% ± 0.0081 for SFII, 27.60% ± 0.0139 for SFIII, and 12.91% ± 0.0045 for SFIV (Table 2). Hypothesis testing showed significant statistical difference between the average and variance of each subfamily except between SFI and SFIV (Additional file 6: Table S4). The average sequence divergence between VSAREP subfamilies (*p*-distance) was 24.11% for SFI and SFII, 56.83% for SFI and SFIII, 47.24% for SFI and SFIV, 57.06% for SFII and SFIII, 45.77% for SFII and SFIV, and 44.52% for SFIII and SFIV. AMOVA analysis of the VSAREP sequences showed 46.16% molecular variation (*P* < 0.001) (16.33 of variance components) within VSAREP subfamilies and 53.84% among VSAREP subfamilies (*P* < 0.001) (19.05 of variance components).

**Table 2:** Summary of nucleotide diversity in each VSAREP subfamily

Subfamily	n	Nucleotide diversity (π)
I	86	0.13002 ± 0.0040
II	38	0.20444 ± 0.0081
III	37	0.27597 ± 0.0139
IV	60	0.12912 ± 0.0045

Number of monomeric repeats sequenced (n) and nucleotide diversity (π) ± SD of each repeated subfamily

### Distribution of VSAREP stDNA sequences in each subfamily

Statistical parsimony network analysis revealed a high level of sequence rearrangement within each VSAREP subfamily. In SFI, the sequence groups of *V. rosenbergi* shared with sequence groups of *V. gouldii* (Additional file 7: Figure S3). For SFII, the sequence groups of *V. komodoensis*, *V. rosenbergi*, and *V. salvadorii* were clustered together, while *V. acanthurus* tended to show clear structuring of the sequence group (Additional file 8: Figure S4). For SFIII, the sequence groups of *V. bengalensis* tended to be the structural group except for two *V. bengalensis* clones (VBE9 and VBE16), which overlapped with the sequence groups of *V. dumerilii* and one *V. acanthurus* clone (VAC8) (Additional file 9: Figure S5). For SFIV, the sequence groups of *V. nebulosus*, *V. salvator sulfur*, *V. salvator macromaculatus* (VSAREP1), *V. salvator ziegleri*, *V. rudicollis*, and *V. salvator macromaculatus* (VSAREP2) shared a complex network (Additional file 10: Figure S6).

### Chromosomal distribution of VSAREP1 and VSAREP2 sequences

**Fig. 3:**
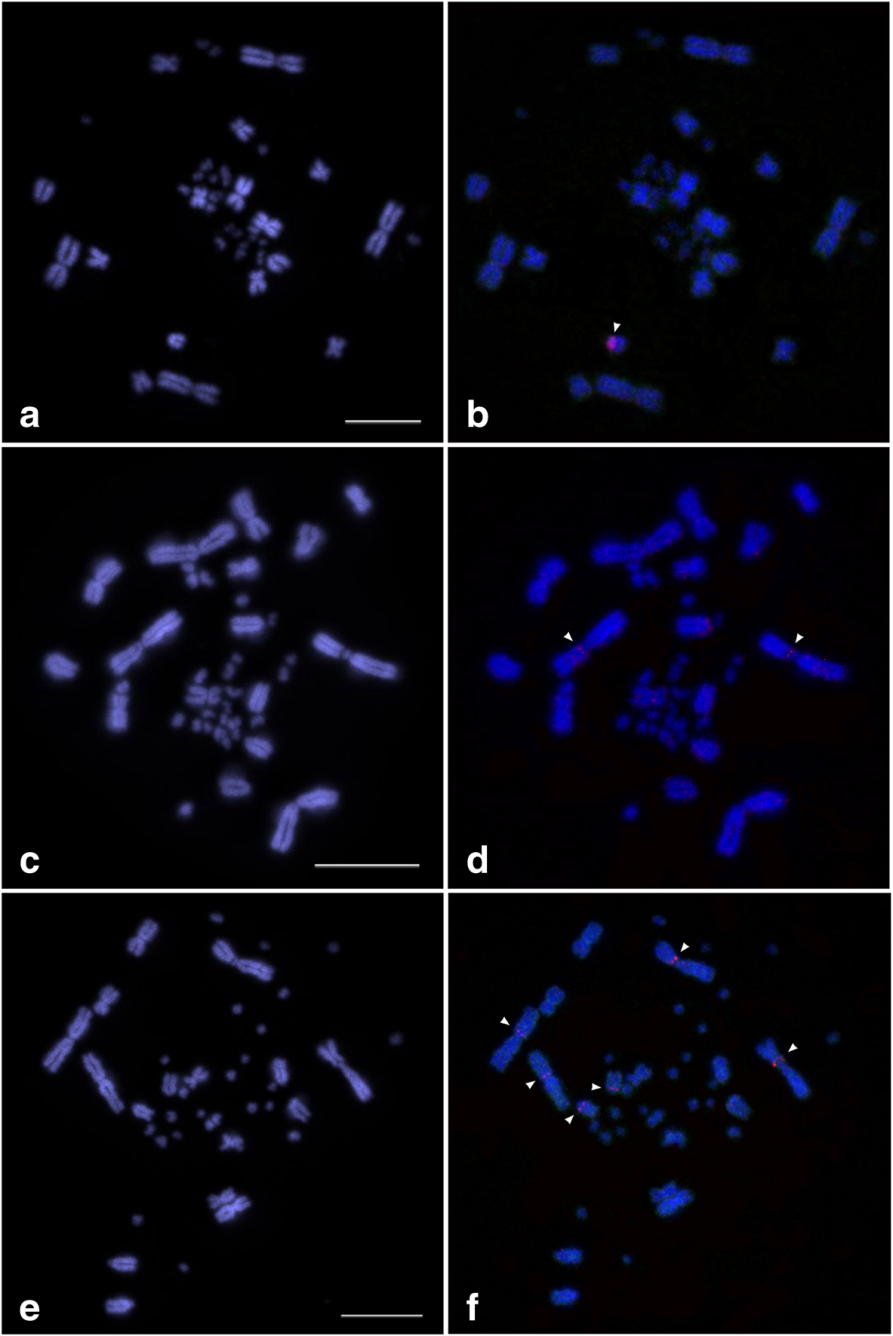
Chromosomal distribution of the VSAREP1 and VSAREP2 sequences on a DAPI-stained metaphase spread prepared from three Australian varanid lizards: *Varanus acanthurus* (**a**, **b**), *V. gouldii* (**c**, **d**), and *V. rosenbergi* (**e**, **f**). Hybridization patterns of Spectrum Orange-labeled VSAREP1 (red) (b, d, f) and SpectrumGreen-labeled VSAREP2 (green) (no signal) on DAPI-stained chromosomes. Fluorescent DAPI-stained pattern of chromosomes are shown in **a**, **c**, and **e**. Arrowheads indicate the hybridization signals. Scale bar represents 10 μm. VSAREP1 sequences were localized to the largest microchromosome in *V. acanthurus*, at the pericentromeric region of chromosome 1p in *V. gouldii*, and at the pericentromeric regions of chromosome 1p and 2p and the centromeric region of chromosome 7 in *V. rosenbergi*

The VSAREP1 sequences were cross-hybridized to chromosomes of the three Australian varanids. VSAREP1 sequences were localized to the largest microchromosome in *V. acanthurus* (Figs. 3a and b). Faint signals of VSAREP1 sequences were observed at the pericentromeric region of chromosome 1p in *V. gouldii* (VGO1p) (Figs. 3c and d), and at the pericentromeric regions of chromosome 1p in *V. rosenbergi* (VRO1p) and VRO2p, and the centromeric region of VRO7 (Figs. 3e and f). No hybridization signal of VSAREP2 was found on chromosomes of the three Australian varanids.

### Chromosomal distribution of VSAREP stDNA sequences isolated from three Australian varanids

**Fig. 4:**
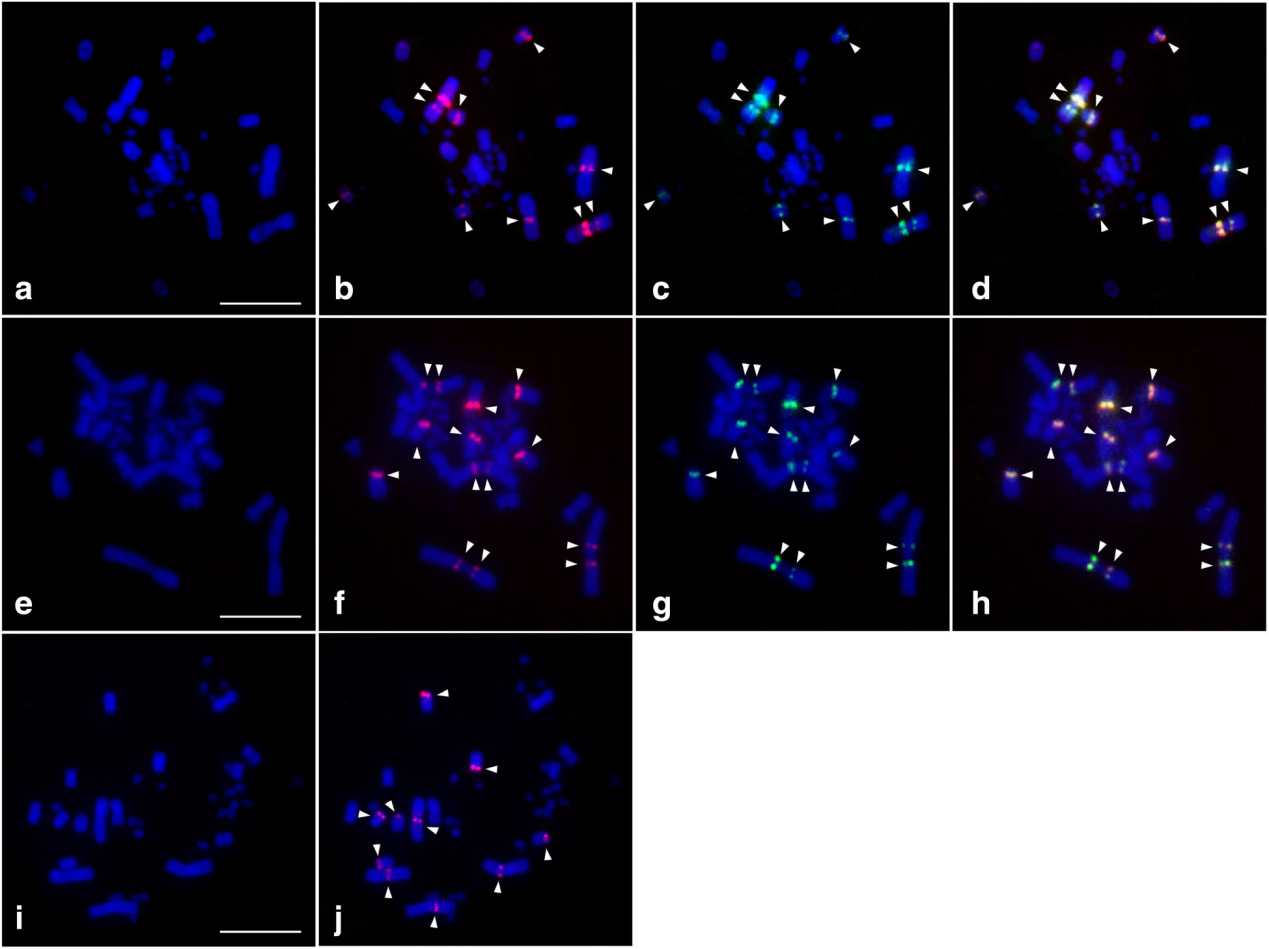
Chromosomal distribution of the VSAREP satellite DNA (stDNA) isolated from each Australian varanids on a DAPI-stained metaphase spread prepared from three Australian varanid lizards: *Varanus acanthurus* (**a** – **d**), *V. rosenbergi* (**e** – **h**), and *V. gouldii* (**i** – **j**). Hybridization patterns of rhodamine-labeled VSAREP stDNA (red) ((*V. acanthurus*, clone no. 3: SFII) **b**, (*V. rosenbergi*, clone no. 14: SFII) **f**, and (*V. gouldii*, clone no. 13: SFI) **j**) or FITC-labeled VSAREP stDNA (green) ((*V. acanthurus*, clone no. 4: SFIII) **c** and (*V. rosenbergi*, clone no. 9: SFI) **g**) and their co-hybridization pattern (**d**, **h**). Fluorescent DAPI-stained pattern of chromosomes are shown in a, e, and i. Arrowheads indicate the hybridization signals. Scale bars represent 10 μm

Five VSAREP stDNA sequences were randomly selected from each subfamily detected in Australian varanids and localized on Australian varanid chromosomes. Clone no. 3 and clone no. 4 from SFII and SFIII, respectively, were mapped on both pericentromeric regions of chromosome 1p in *V. acanthurus* (VAC1p), VAC1q, VAC2q, and the centromeric region of VAC7 and VAC8 (Figs. 4a – d). Clone no. 13 from SFI was localized to the pericentromeric region of VGO1q, VGO2p, and the centromeric regions of VGO5, VGO6 and VGO7 (Figs. 4i and j). Additionally, clone no. 14 and 9 from SFI and SFII, respectively were located in the pericentromeric regions of VRO1p, VRO1q, VRO2p and VRO2q, and the centromeric regions of VRO5, VRO6 and VRO7 (Figs. 4e – h).

## Discussion

### Evolution of varanids based on VSAREP stDNA family

Molecular phylogenetic studies using nuclear functional genes (*BDNF*: brain-derived neurotrophic factor, *BMP*: bone morphogenetic protein, and *NT3*: neurotrophin-3), and mitochondrial genes (*ND1*: NADH dehydrogenase 1, *ND2*: NADH dehydrogenase 2, *COI*: cytochrome C oxidase subunit I, tRNAs: transfer RNAs, and O_L_: origin of light-strand replication) suggest an Asian origin of varanids followed by dispersal to Africa 49–33 million years ago (MYA) and then to Australia in the Late Eocene–Oligocene 39–26 MYA [58, 59]. However, an alternative hypothesis suggesting an African origin, followed by dispersal to Asia and Australia remains controversial [60]. Sequence conservation of the VSAREP stDNA family was examined in 16 varanids, except for *V. rosenbergi*, due to the insufficient amount of genomic DNA available for this species. Dot-blot analysis using VSAREP1 and VSAREP2 showed a clear positive hybridization signal in Asian and Australian varanids, but not in African varanids. This suggests that the copy number of VSAREP in African varanids may be too few to be detected by dot-blots. The absence of VSAREP was also found in other squamate reptiles [7], collectively suggesting that the VSAREP was acquired in the genome of the common ancestor of Asian and Australian varanids. The sequences were then amplified independently after they diverged from African varanids. This supports the hypothesis of an African origin of varanids [60]. On the contrary, the loss of VSAREP in African varanids might result from a stochastic effect due to random genetic drift. Alternatively, large stDNA sequence divergences can often be observed among related species such as pupfish, fishes from the family Sparidae, and the *Drosophila obscura* group whose stDNAs were arisen around 42–2 MYA [18, 61, 62]. VSAREP emerged at least 40 MYA according to the divergence of varanid lineage [59]. This divergence time is, therefore, long enough for sequence differentiation in the African varanid lineage. These two pieces of evidence tally with the hypothesis of Asian varanid origin, and the VSAREP may be replaced by other stDNA sequences with low sequence similarity to VSAREP in African varanids.

Intriguingly, comparison of VSAREP sequences revealed average sequence similarity of 80% between Asian and Australian varanids. This result suggests the presence of ancestral repeated variants, or a recent common ancestor in Asian and Australian varanids. All VSAREP sequences were GC-rich as also found in *Eumeces schneideri* [63], differing with stDNA of other squamate reptiles as AT-rich [8, 39–44, 64]. The conserved sequence motifs of VSAREP stDNA families were found in all sequence units, but no significant similarity was found with other sequences deposited in databases. Structural and functional studies are required to explain this molecular mechanism. Putative secondary structures were found in all VSAREP sequences. This might be important for chromatin condensation, or the interaction between protein and DNA [14, 65, 66], and suggests that VSAREPs contain common structural features of stDNA which were retained in Asian and Australian varanid genomes under selective pressure.

### Diversity of VSAREP stDNA subfamilies

Sequence divergences of VSAREP were mainly caused by nucleotide substitutions, while indels were rarely found in sequences of Asian and Australian varanids. Molecular phylogeny, based on concatenated sequences of nuclear and mitochondrial functional genes revealed that Asian and Australian varanids diverged from African varanids around 49–33 MYA [59]. This time period implies a substitution rate for *BDNF* of 0.0007% (± 0.000014) per million year (MY), *NT3* of 0.000594% (± 0.000013) per MY, *BMP* of 0.000574% (± 0.000021) per MY, mitochondrial *ND1* – *ND2* of 0.0031% (± 0.00026) per MY; however, an evolutionary rate of 0.0043% (± 0.00017) per MY was higher in the VSAREP family. AMOVA analysis indicated that molecular variation was more likely distributed between species than within species, but comparison of VSAREP sequences revealed a higher degree of intraspecific sequence divergences (9.41–26.46%) than those of interspecific divergences (0–15.60%). This incongruity might result from the number of sequences analyzed that differed among species, leading to variance bias. By contrast, phylogenetic analysis of VSAREP revealed four VSAREP subfamilies of VSAREP stDNA, each showing a high level of sequence divergence. The similarity of each unit of VSAREP with the same subfamily from different species was higher than those of other subfamilies belonging to the same species. AMOVA analysis also indicated differentiation between VSAREP subfamilies. This agreed with the library model of stDNA evolution [67], in which different stDNA families or subfamilies coexist in the genomes of related species and are amplified differentially among species [68–71]. This suggests that nucleotide substitutions might accumulate more slowly than homogenization rates in each subfamily, resulting in the absence of species-specific stDNA profiles. VSAREP sequences, therefore, may not be ideal for varanid identification.

The complex network pattern indicates rearrangements of sequence variants in all VSAREP subfamilies. However, no structuring of sequence groups at the species level in SFI or SFIV was found, while the tendency of the sequence group of *V. acanthurus* (SFII) and *V. bengalensis* (SFIII) was the structuring. Different average sequence divergence within VSAREP subfamilies is statistically supported for most VSAREP subfamilies, but not between SFI and SFIV (Additional files 6: Table S4). This suggests that VSAREP sequences in SFI and SFIV differentiated with a high homogenization rate in each subfamily (Additional file 7: Figure S3 and Additional file 10: Figure S6).

Most VSAREP sequences shared among Asian varanids in SFIV contained sequences 190 bp in length, though one insertion of C or A was found in *V. nebulosus* (191 bp). However, smaller sizes (185–187) of VSAREP were also found in SFIV which contained 5–6 bp deletion. This 5–6 bp deletion was found in SFIII for *V. dumerilii* and *V. bengalensis*, except for one clone from *V. acanthurus* (194 bp). This suggests that homogenization with 5–6 bp deletion became fixed in SFIII. According to molecular phylogeny [59], *V. dumerilii* is likely a sister to *V. salvator macromaculatus* and *V. rudicollis*; this suggests that smaller sizes of VSAREP were considered as ancestral sequences. The 190 and 191 bp monomer repeats probably derived from a 5–6 bp insertion that occurred in VSAREP2 repeats belonging to SFIV. Extensive diversification was found in SFI (*V. rudicollis* and *V. gouldii*) and SFII (*V. komodoensis*, *V. rosenbergi*, *V. acanthurus*, and *V. salvadorii*), which contained unit size of 191 bp. This was also found in *V. nebulosus* (SFIV), although sequence divergence among SFI, SFII, and SFIV were not low. This suggests that VSAREP evolved gradually through nucleotide substitution and rapid amplification in each VSAREP subfamily.

### Chromosomal distribution of VSAREP stDNA subfamilies in Australian varanids

In Asian varanids, VSAREP1 was localized to the pericentromeric region of chromosome 1q in *V. salvator macromaculatus* (VSA(M)1q) and VSA(M)2q, the centromeric region of VSA(M)5, and 3 pairs of microchromosomes in *V. salvator macromaculatus* [7]. However, the chromosomal distribution of VSAREP1 differed among three Australian varanids and also in the Asian varanids. This suggests that VSAREP1 was dispersed in the ancestral genome of Australian varanids and subsequently amplified on different chromosomes independently in each species, consistent with the library model [67]. The loss or gain of copy number on different chromosomes in Australian varanids resulted from unequal crossovers between sister chromatids or intra- and interchromosomal recombination. No VSAREP2 was observed on the three Australian varanid chromosomes, which suggests that the copy numbers of VSAREP2 may be too few for detection by FISH mapping. Alternatively, faint signals of VSAREP1 were observed on the most Australian varanids. This might be a consequence of a cross-hybridization with other monomer variants that also escaped detection with VSAREP2.

**Fig. 5:**
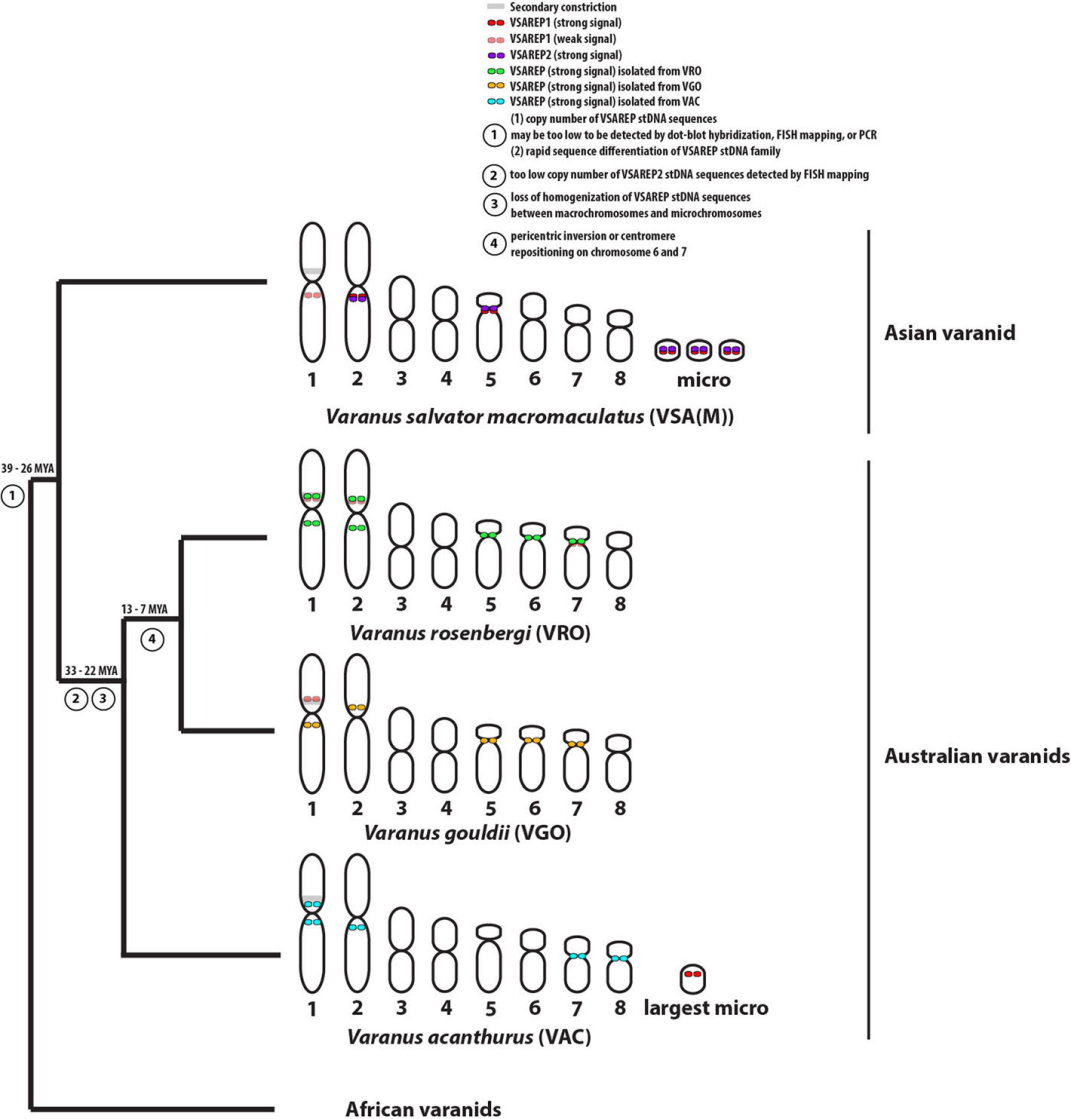
Schematic representation for karyotype and VSAREP satellite DNA (stDNA) chromosomal distribution in varanids. Phylogeny was partially derived from Vidal et al. [59]. Divergence times were estimated in million years ago (MYA) for each node [59]. Karyotype of *Varanus salvator macromaculatus* (VSA(M)) was obtained from Chaiprasertsri et al. [7] and Srikulnath et al. [35] and karyotypes of *V. acanthurus* (VAC), *V. gouldii* (VGO), and *V. rosenbergi* (VRO) were obtained from Matsubara et al. [36]. FISH indicates fluorescence in situ hybridization, and PCR indicates polymerase chain reaction

By contrast, different VSAREP subfamilies were mapped on the same chromosomal location in each Australian varanid. However, these repeats were found in different chromosomal regions of chromosomes 6–8 among the three Australian varanids, whose chromosome morphologies differed as submetacentric or acrocentric chromosomes. Srikulnath et al. [35] asserted that within varanid karyotypes, the variation occurred only in the morphology of the macrochromosomes, in particular chromosomes 6–8, resulting from pericentric inversion or centromere repositioning as observed in the cytogenetic maps of *V. salvator macromaculatus* and *V. exanthematicus*. This suggests that the changes in the stDNA locations correlated with chromosomal rearrangements, leading to karyotypic differences among the three Australian varanids (Fig. 5).

### Different subfamilies of VSAREP stDNA related to chromosome size-correlated compartmentalization in varanids

stDNA sequences have been proved to be significant molecular cytogenetic markers to decipher genomic compartmentalization in karyotypes of many birds and reptiles [7, 8, 72–78]. However, no macro- or microchromosome specific centromeric stDNA sequences have been isolated in squamate reptiles. This suggests that homogenization of centromeric stDNA sequences between macro- and microchromosomes is a general characteristic of squamate reptiles, as compared with turtles and birds where both chromosome-sized specific and non-specific centromeric stDNA sequences were found [72–78]. Interestingly, VSAREP isolated from the three Australian varanids was specifically located in the pericentromeric or centromeric regions of the macrochromosomes. Therefore, chromosome size-correlated compartmentalization between macro- and microchromosomes possibly occurred in the centromeric stDNA sequences of the three Australian varanids as the first case found in squamate reptiles. In spite of the same stDNA family, VSAREP1 and VSAREP2 were mapped on both macro- and microchromosomes in *V. salvator macromaculatus*. The disappearance of the VSAREP stDNA localization on microchromosomes of Australian varanids was probably caused by the loss of copy number, resulting from non-homologous recombination or rapid amplification of the new subfamily on the macrochromosomes. However, further study is required to fully comprehend the evolutionary process of chromosome size-correlated compartmentalization at molecular level in varanids and squamates in general.

Sequence analysis and chromosomal mapping enabled us to delineate the evolutionary origin and diversification of VSAREP stDNA. Homogenization of VSAREP stDNA appeared independently in each Asian and Australian varanid lineage, leading to the absence of species-specific stDNA sequences. This stDNA family also correlates with chromosomal rearrangements and chromosome size-correlated compartmentalization in the varanid lineage. Whole genome sequencing and transcriptomic analysis of varanids are required to investigate structural and functional studies of DNA-protein interactions, to further explain the potential molecular mechanism of VSAREP for genome organization of varanids and squamate reptiles.

## Conclusions

VSAREP stDNA is conserved in the genome of both Asian and Australian varanids and shared within the four VSAREP subfamilies. This suggests that VSAREP stDNA families lack homogenized species-specific nucleotide positions in varanid lineage, resulting in non-species-specific evolution of stDNA profiles. VSAREP stDNA sequences were located on both macro- and microchromosomes in the Asian varanid (*V. salvator macromaculatus*), but not for the three Australian varanids, with VSAREP specifically located on macro- or microchromosomes (Fig. 5). This suggests that chromosome size-correlated compartmentalization occurred in the three Australian varanids. Moreover, changes in location of VSAREP stDNA in each Australian varanid suggest a correlation with chromosomal rearrangements, leading to karyotypic differences among these species.

## Additional files

Additional file 1: Table S1. Fluorescence in situ hybridization mapping used randomly selected VSAREP clones from each VSAREP subfamily isolated from genomic DNA of three Australian varanids (*Varanus acanthurus*, *V. gouldii*, and *V. rosenbergi*). (DOC 35 kb)

Additional file 2: Figure S1. Secondary structures of VSAREP satellite DNA (stDNA) family of 12 varanids were formed using RNAfold web server (http://rna.tbi.univie.ac.at/cgi-bin/RNAWebSuite/RNAfold.cgi) [46]. VSAREP stDNA sequences of 12 varanids were used: *Varanus salvator macromaculatus* (VSA(M)), *V. salvator sulfur* (VSA(S)), *V. salvator ziegleri* (VSA(Z)), *V. bengalensis* (VBE), *V. nebulosus* (VNE), *V. rudicollis* (VRU), *V. dumerilii* (VDU), *V. salvadorii* (VSALV), *V. komodoensis* (VKO), *V. rosenbergi* (VRO), *V. gouldii* (VGO), and *V. acanthurus* (VAC). SF indicates repeated subfamily. The putative secondary structures are often found in stDNA sequences, including VSAREP sequences. (TIFF 3393 kb)

Additional file 3: Figure S2. Multiple alignment of all VSAREP satellite DNA (stDNA) sequences from the consensus sequences of each species. VSAREP stDNA sequences of 12 varanids were used: *Varanus salvator macromaculatus* (VSA(M)), *V. salvator sulfur* (VSA(S)), *V. salvator ziegleri* (VSA(Z)), *V. bengalensis* (VBE), *V. nebulosus* (VNE), *V. rudicollis* (VRU), *V. dumerilii* (VDU), *V. salvadorii* (VSALV), *V. komodoensis* (VKO), *V. rosenbergi* (VRO), *V. gouldii* (VGO), and *V. acanthurus* (VAC). SF indicates repeated subfamily. The conserved sequence motifs of VSAREP stDNA family are “TGACCCGCGGGTCAGC” and “TTTTBGGCATTTTG” found in all sequence units. The 5–6 bp deletion was found in *V. dumerilii* and *V. bengalensis* of VSAREP subfamily III and VSAREP2 of *V. salvator macromaculatus*. (TIFF 2288 kb)

Additional file 4: Table S2. Summary of repeat units and subfamilies in each species. (DOC 46 kb)

Additional file 5: Table S3. Pairwise comparison of VSAREP satellite DNA sequence divergences among 12 varanids. (DOC 64 kb)

Additional file 6: Table S4. T-test and F-test analyses using the average and standard deviation of nucleotide diversity of each VSAREP subfamily. (DOC 50 kb)

Additional file 7: Figure S3. Statistical parsimony network of VSAREP subfamily I constructed from all VSAREP sequence units of *Varanus gouldii* (VGO) and *V. rosenbergi* (VRO). (TIFF 813 kb)

Additional file 8: Figure S4. Statistical parsimony network of VSAREP subfamily II constructed from all VSAREP sequence units of *Varanus rosenbergi* (VRO), *V. komodoensis* (VKO), *V. acanthurus* (VAC), and *V. salvadorii* (VSALV). (TIFF 7049 kb)

Additional file 9: Figure S5. Statistical parsimony network of VSAREP subfamily III constructed from all VSAREP sequence units of *Varanus acanthurus* (VAC), *V. dumerilii* (VDU), and *V. bengalensis* (VBE). (TIFF 1141 kb)

Additional file 10: Figure S6. Statistical parsimony network of VSAREP subfamily IV constructed from all VSAREP sequence units of *Varanus salvator macromaculatus* (VSA(M)) comprising VSAREP1 and VSAREP2, *V. salvator sulfur* (VSA(S)), *V. salvator ziegleri* (VSA(Z)), *V. nebulosus* (VNE), and *V. rudicollis* (VRU). (TIFF 2542 kb)

## Abbreviations

*BDNF*: Brain-derived neurotrophic factor
BI: Bayesian inference
*BMP*: Bone morphogenetic proteins
*COI*: Cytochrome C oxidase subunit I
DAPI: 4′,6-diamidino-2-phenylindole
DDBJ: DNA Data Bank of Japan
EDTA: Ethylenediaminetetraacetic acid
indels: Insertions and deletions
MY: Million year
MYA: Million years ago
*ND1*: NADH dehydrogenase 1
*ND2*: NADH dehydrogenase 2
*NT3*: Neurotrophin-3
O_L_: Origin of light-strand replication
PCR: Polymerase chain reaction
*p*-distances: Pairwise distances
SDS: Sodium dodecyl sulfate
SF: Subfamily
SSC: Saline-sodium citrate
stDNAs: Satellite DNAs
tRNAs: Transfer RNAs
VAC: *Varanus acanthurus*
VBE: *Varanus bengalensis*
VDU: *Varanus dumerilii*
VEX: *Varanus exanthematicus*
VGO: *Varanus gouldii*
VGR: *Varanus griseus*
VJO: *Varanus jobiensis*
VKO: *Varanus komodoensis*
VNE: *Varanus nebulosus*
VNI: *Varanus niloticus*
VOB: *Varanus obor*
VRO: *Varanus rosenbergi*
VRU: *Varanus rudicollis*
VSA(M): *Varanus salvator macromaculatus*
VSA(S): *Varanus salvator sulfur*
VSA(Z): *Varanus salvator ziegleri*
VSALV: *Varanus salvadorii*
π value: Nucleotide diversity
