# Supplementary material for: Lack of satellite DNA species-specific homogenization and relationship to chromosomal rearrangements in monitor lizards (Varanidae, Squamata)
Source: BMC Evol Biol. 2017 Aug 16;17:193. doi: 10.1186/s12862-017-1044-6 (PMC5559828; doi:10.1186/s12862-017-1044-6)
Supplement: Supplementary file 1 — Fluorescence in situ hybridization mapping used randomly selected VSAREP clones from each VSAREP subfamily isolated from genomic DNA of three Australian varanids (Varanus acanthurus, V. gouldii, and V. rosenbergi). (DOC 35 kb) [file 12862_2017_1044_MOESM1_ESM.doc]

Table S1. Fluorescence *in situ* hybridization mapping used randomly selected VSAREP clones from each VSAREP subfamily isolated from genomic DNA of three Australian varanids (*Varanus acanthurus*, *V. gouldii*, and *V. rosenbergi*).

| Species | Clone no. | Subfamily^a^ | Unit |
| --- | --- | --- | --- |
| *Varanus acanthurus* | 3 | SFII | VAC6 – VAC7 |
|  | 4 | SFIII | VAC8 |
| *Varanus gouldii* | 13 | SFI | VGO6 – VGO8 |
| *Varanus rosenbergi* | 9 | SFII | VRO23 – VRO25 |
|  | 14 | SFI | VRO39 – VRO42 |

^a^SF indicates VSAREP subfamily
