# Supplementary material for: Lack of satellite DNA species-specific homogenization and relationship to chromosomal rearrangements in monitor lizards (Varanidae, Squamata)
Source: BMC Evol Biol. 2017 Aug 16;17:193. doi: 10.1186/s12862-017-1044-6 (PMC5559828; doi:10.1186/s12862-017-1044-6)
Supplement: Supplementary file 4 — Summary of repeat units and subfamilies in each species. (DOC 46 kb) [file 12862_2017_1044_MOESM4_ESM.doc]

Table S2. Summary of repeat units and subfamilies in each species.

| Species | Subfamily^a^ | n^b^ | Unit |
| --- | --- | --- | --- |
| *Varanus salvator macromaculatus* | SFIV | 10 | VSA(M)1—VSA(M)10 |
| *Varanus salvator sulfur* | SFIV | 14 | VSA(S)1—VSA(S)14 |
| *Varanus salvator ziegleri* | SFIV | 6 | VSA(Z)1—VSA(Z)6 |
| *Varanus bengalensis* | SFIII | 16 | VBE1—VBE16 |
| *Varanus nebulosus* | SFIV | 9 | VNE1—VNE9 |
| *Varanus rudicollis* | SFIV | 21 | VRU1—VRU21 |
| *Varanus dumerilii* | SFIII | 20 | VDU1—VDU20 |
| *Varanus salvadorii* | SFII | 2 | VSALV1—VSALV2 |
| *Varanus komodoensis* | SFII | 21 | VKO1—VKO21 |
| *Varanus rosenbergi* | SFII | 3 | VRO23—VRO25 |
|  | SFI | 53 | VRO1—VRO22, VRO26—VRO56 |
| *Varanus gouldii* | SFI | 33 | VGO1—VGO33 |
| *Varanus acanthurus* | SFII | 12 | VAC1—VAC7, VAC9—VAC13 |
|  | SFIII | 1 | VAC8 |

^a^SF indicates VSAREP subfamily

^b^Number of monomeric repeats sequenced (n)
