# Supplementary material for: Lack of satellite DNA species-specific homogenization and relationship to chromosomal rearrangements in monitor lizards (Varanidae, Squamata)
Source: BMC Evol Biol. 2017 Aug 16;17:193. doi: 10.1186/s12862-017-1044-6 (PMC5559828; doi:10.1186/s12862-017-1044-6)
Supplement: Supplementary file 5 — Pairwise comparison of VSAREP satellite DNA sequence divergences among 12 varanids. (DOC 64 kb) [file 12862_2017_1044_MOESM5_ESM.doc]

Table S3. Pairwise comparison of VSAREP satellite DNA sequence divergences among 12 varanids.

|  | VAC | VBE | VDU | VGO | VKO | VNE | VRO | VRU | VSA(M) | VSA(S) | VSALV | VSA(Z) |
| --- | --- | --- | --- | --- | --- | --- | --- | --- | --- | --- | --- | --- |
| *Varanus acanthurus* (VAC) |  |  |  |  |  |  |  |  |  |  |  |  |
| *Varanus bengalensis*  (VBE) | 0.127 |  |  |  |  |  |  |  |  |  |  |  |
| *Varanus dumerilii* (VDU) | 0.119 | 0.033 |  |  |  |  |  |  |  |  |  |  |
| *Varanus gouldii* (VGO) | 0.084 | 0.156 | 0.143 |  |  |  |  |  |  |  |  |  |
| *Varanus komodoensis* (VKO) | 0.069 | 0.128 | 0.120 | 0.068 |  |  |  |  |  |  |  |  |
| *Varanus nebulosus* (VNE) | 0.122 | 0.111 | 0.086 | 0.119 | 0.120 |  |  |  |  |  |  |  |
| *Varanus rosenbergi* (VRO) | 0.064 | 0.129 | 0.120 | 0.014 | 0.046 | 0.118 |  |  |  |  |  |  |
| *Varanus rudicollis* (VRU) | 0.126 | 0.096 | 0.076 | 0.131 | 0.131 | 0.022 | 0.129 |  |  |  |  |  |
| *Varanus salvator macromaculatus* (VSA(M)) | 0.117 | 0.095 | 0.079 | 0.110 | 0.110 | 0.006 | 0.111 | 0.012 |  |  |  |  |
| *Varanus salvator sulfur* (VSA(S)) | 0.123 | 0.112 | 0.089 | 0.118 | 0.117 | 0.000 | 0.114 | 0.029 | 0.012 |  |  |  |
| *Varanus salvadorii* (VSALV) | 0.071 | 0.141 | 0.133 | 0.077 | 0.063 | 0.139 | 0.052 | 0.141 | 0.133 | 0.131 |  |  |
| *Varanus salvator ziegleri* (VSA(Z)) | 0.128 | 0.123 | 0.093 | 0.120 | 0.128 | 0.004 | 0.121 | 0.040 | 0.023 | 0.007 | 0.142 |  |
