# Supplementary material for: Lack of satellite DNA species-specific homogenization and relationship to chromosomal rearrangements in monitor lizards (Varanidae, Squamata)
Source: BMC Evol Biol. 2017 Aug 16;17:193. doi: 10.1186/s12862-017-1044-6 (PMC5559828; doi:10.1186/s12862-017-1044-6)
Supplement: Supplementary file 6 — T-test and F-test analyses using the average and standard deviation of nucleotide diversity of each VSAREP subfamily. (DOC 50 kb) [file 12862_2017_1044_MOESM6_ESM.doc]

Table S4. T-test and F-test analyses using the average and standard deviation of nucleotide diversity of each VSAREP subfamily.

|  | T-test | | | | | | F-test | | | | |
| --- | --- | --- | --- | --- | --- | --- | --- | --- | --- | --- | --- |
| Pairwise | µ1±SD1 | µ2±SD2 | df | *P* value | Difference estimate | Summary | SD1 | SD2 | *P* value | Difference estimate | Summary |
| SFI-SFII | 0.13002±0.0040 | 0.20444±0.0081 | 45 | 0.000 | 0.07442 | SFII > SFI | 0.0040 | 0.0081 | 0.000 | 5.82890 | SFI ≠ SFII |
| SFI-SFIII | 0.13002±0.0040 | 0.27597±0.0139 | 38 | 0.000 | 0.14595 | SFIII > SFI | 0.0040 | 0.0139 | 0.000 | 14.11500 | SFI ≠ SFIII |
| SFI-SFIV | 0.13002±0.0040 | 0.12912±0.0045 | 144 | 0.206 | -0.00090 | SFI = SFIV | 0.0040 | 0.0045 | 0.112 | 1.45530 | SFI = SFIV |
| SFII-SFIII | 0.20444±0.0081 | 0.27597±0.0139 | 57 | 0.000 | -0.07153 | SFIII > SFII | 0.0081 | 0.0139 | 0.000 | 0.41295 | SFII ≠ SFIII |
| SFII-SFIV | 0.20444±0.0081 | 0.12912±0.0045 | 51 | 0.000 | 0.07532 | SFII > SFIV | 0.0081 | 0.0045 | 0.000 | 4.00530 | SFII ≠ SFIV |
| SFIII-SFIV | 0.27597±0.0139 | 0.12912±0.0045 | 95 | 0.000 | -0.14685 | SFIII > SFIV | 0.0139 | 0.0045 | 0.000 | 0.10310 | SFIII ≠ SFIV |

SF indicates repeated subfamily
